# The Impact of Mothers’ Perceived Unsupportive Intergenerational Co-Parenting on Children’s Social Competence: Evidence from China

**DOI:** 10.3390/ijerph20010427

**Published:** 2022-12-27

**Authors:** Xinpei Xu, Lihong Song, Xiaoyun Li, Yan Li

**Affiliations:** Shanghai Institute of Early Childhood Education, Shanghai Normal University, Shanghai 200234, China

**Keywords:** intergenerational co-parenting, parenting style, social competence, psychological flexibility

## Abstract

This study examined whether parenting styles mediated the relationship between unsupportive intergenerational co-parenting and children’s social competence, and whether the first stage of the mediating process, as well as the direct association between unsupportive intergenerational co-parenting and children’s social competence, was moderated by maternal psychological flexibility. The theoretical model was tested using data collected from 412 mothers of children aged 3–6 years at four kindergartens in Shanghai, China. The results showed that: (1) unsupportive intergenerational co-parenting was negatively associated with children’s social competence through decreased maternal authoritative parenting and increased authoritarian parenting and (2) the first stage of the mediation mechanism was moderated by maternal psychological flexibility. Specifically, unsupportive intergenerational co-parenting was significantly associated with authoritative and authoritarian parenting for mothers with low and high psychological flexibility, respectively, and the magnitude of the association was higher for mothers with low psychological flexibility. These findings extend the understanding of how and when unsupportive intergenerational co-parenting impacts children’s social competence.

## 1. Introduction

Social competence (SC) refers to the ability to coordinate behavioral, cognitive, and emotional resources actively and skillfully in a specific context to achieve social goals while maintaining harmony with others [1]. Prior studies have shown that high degrees of SC in children are associated with higher peer ratings based on likability [2], as well as reduced behavioral problems [3]. SC is commonly used as an indicator of children’s social adjustment [4,5]. During early childhood, the family serves as the main microsystem and has a crucial impact on children’s social adjustment [6]. Based on family systems theory [7], the involvement of adult caregivers, aside from the mother (e.g., the father, grandparents, and other relatives), extends the mother-and-child connection into a tripartite relationship. Further, children benefit when both sides of the family involved in parenting develop a cohesive relationship that balances the family dynamic. Thus, SC among children may be correlated with the quality of the co-parenting relationship between primary caregivers.

Intergenerational dependence increases with an aging population [8]. An increasing number of children are living with their parents and one or more grandparents [9]. Grandparents play a central and active role as primary caregivers for a child [10]. In the Netherlands, the proportion of parents who involved grandparents in childcare increased from 23% in 1992 to 66% in 2015 [11]. In 2015, approximately 7.3 million grandparents in the United States (U.S.) lived with their grandchildren, and more than 35% of them were their grandchild’s primary caregiver [12]. Further, in China, a survey report showed that grandparents were involved in raising grandchildren aged 3–6 years in 72.9% of urban households [13]. Thus, grandparents’ involvement in raising grandchildren is a widespread phenomenon.

Recently, researchers have increased their focus on children’s social adjustment in three-generation families due to the prevalence of grandparents’ involvement in early childhood parenting. This contrasts with previous studies that have examined children’s social adjustment and the associated individual effects of grandparents’ involvement [14,15]. Limited studies have examined the quality of intergenerational co-parenting (IC); thus, whether grandparents’ involvement is beneficial or harmful to children’s social adjustment remains to be established [16]. Further, researchers have only explored the direct impact of IC on children’s social adjustment, and have neglected the underlying mechanisms. According to ecological model of co-parenting [17], co-parenting can directly and indirectly affect children’s development [18]. Moreover, parental characteristics, such as psychological flexibility, may influence how parents feel about and respond to harmonious or conflicting co-parenting, and may play a moderating role in co-parenting, parenting behavior, and child adjustment. Thus, this study aims to expand the research by examining the mediating role of parenting styles and the moderating role of maternal psychological flexibility in the relationship between IC and children’s SC. Because parents often experience conflict with grandparents during the childcare process in Chinese families [19], this study is mainly interested in unsupportive IC.

### 1.1. The Relation between IC and Children’s SC

IC pertains to the co-parenting of children between parents and grandparents [20,21]. Grandparents’ involvement in child-rearing is a main source of social support for parents and plays a crucial role in the care and socialization of children [22]. However, conflicts often arise regarding parenting issues between the parents and grandparents of the child due to differences in parenting beliefs and styles [23]. Conflicting relationships are more likely to yield unsupportive co-parenting between parents and grandparents, which can include competing to attract the child’s attention, criticizing actions or statements, or interrupting individual interactions with the child [24].

The interaction between parents and grandparents during co-parenting provides a social learning environment for children. Social learning theory [25] suggests that children observe and imitate adults’ interactive behaviors and apply them to their peer interactions. Thus, conflict in the IC relationship sets a negative precedent for children. Children who grow up in families with higher levels of intergenerational conflict may lack positive emotion regulation strategies and exhibit more problematic behaviors. Buckingham-Howes et al. [26] found that mother–grandmother conflict during the first 24 months of a child’s life is associated with elevated levels of externalizing behaviors by age seven. Further, Barnett et al. [27] revealed that verbal conflicts between the mother and grandmother are related to fewer prosocial behaviors and more problem behaviors among children.

Prior studies have examined IC in the specific context of child-rearing as opposed to general relational conflict and children’s overall social adjustment. For example, Barnett et al. [27] found that mother–grandmother co-parenting cooperation in low-income families was associated with high levels of children’s SC. Further studies revealed that harmonious IC was related to improved social development [16,28]. The extant research has also focused on co-parenting cooperation by measuring the extent to which co-parents work collaboratively to raise their children, and some studies have considered co-parenting as a latent variable that is represented by multiple affective and behavioral dimensions. However, limited studies have examined the individual influence of unsupportive IC on children’s SC. Therefore, this study focuses on unsupportive IC and its relationship with children’s SC in order to contribute empirical evidence to the extant research.

### 1.2. The Mediating Role of Parenting Styles

Parenting styles refer to the relatively stable behavior patterns that parents exhibit in the process of bonding with their children, and comprise the various attitudes, values, and emotional atmospheres expressed by parents toward their children [29]. Authoritative and authoritarian parenting are two typical parenting styles proposed by Baumrind [30] that have been extensively examined in Chinese parenting research [31]. Authoritative parenting is generally viewed as positive and democratic, and is characterized by a considerable amount of warmth, responsiveness, and encouragement toward children’s autonomy [30]. Conversely, authoritarian parenting is viewed as negative and domineering, and is characterized by a combination of low responsiveness and high coercive control. Parents who lack warmth and acceptance toward their children limit their children’s autonomy, demand absolute obedience, and often use coercive discipline strategies such as corporal punishment and verbal hostility [30]. Previous studies have demonstrated that authoritative and authoritarian parenting, respectively, are positively and negatively linked to children’s SC [32,33,34].

In line with the family systems theory [7], co-parenting and parenting are distinct but interrelated subsystems in a family [35]. Growing evidence from Western and Chinese studies has suggested that co-parenting affects parenting styles [36,37]. However, most studies have limited this effect to co-parenting between couples; there is limited information available for the relationship between IC and parenting styles. Xu et al. [38] highlight the impact of perceived social support on Chinese mothers’ parenting styles and reveal that mothers’ perceived social support to be correlated with low authoritative and high authoritarian parenting. Unsupportive IC (i.e., lack of social support) may also be related to parenting styles.

Parenting styles may play a mediating role in the relationship between IC and children’s SC. The prior studies on the family systems theory, such as the ecological model of co-parenting [17], have emphasized that co-parenting can directly and indirectly affect children’s development through parenting styles. Several studies have provided empirical evidence to substantiate this view to a certain extent. For example, Wang et al. [18] revealed that IC was indirectly related to children’s cognitive flexibility through maternal parenting. Li and Liu [16] showed that mothers’ self-efficacy, which is positively correlated with warm and positive controlling parenting behaviors [39,40], mediated the association between IC and children’s SC. However, to date, no studies have directly analyzed the relationship between IC, parenting styles, and children’s SC. Thus, this study explores the mediating role of parenting styles in the relationship between unsupportive IC and children’s SC. This study asserts that unsupportive IC influences parenting styles and results in less authoritative parenting and more authoritarian parenting, which, in turn, are linked to low levels of children’s SC.

### 1.3. The Moderating Role of Parents’ Psychological Flexibility

As previously mentioned, the family systems theory asserts that varying degrees of family functioning are interrelated. Thus, conflict in one subsystem (e.g., within the parent–grandparent dyad) can spill over into other subsystems in the family (e.g., the parent–child dyad), leading to adverse parenting behaviors and children’s psychosocial ill-being. While unsupportive IC can negatively affect parenting styles and children’s SC [16,18,28], its effect may vary based on parents’ psychological flexibility.

Psychological flexibility refers to the ability to comprehensively interact with a current external situation, be consciously aware of the current internal mind state (i.e., memory, thoughts, emotions, motivation, and other mental activities), and actively maintain or change behaviors based on certain values [41]. Studies have found that more psychological flexibility among parents is related to less negative and inconsistent parenting practices [42], decreased parenting stress [43], and increased psychosocial well-being in children [44].

According to the vulnerability stress adaptation model (VSA) [45], in the face of a stressful event or difficult situation, two people in an intimate relationship need to use internal qualities, such as psychological flexibility, interpersonal skills, and communication, to deal with it. Failure to properly handle such an event or situation may undermine the quality of the relationship and create additional stressful events, such as increased levels of conflict in the future [46]. The transactional theory of stress and coping [47] also emphasizes the dynamic relationship between individuals and stressful environments. Specifically, individuals who experience stress in an unfavorable environment explain and evaluate the situation, develop and select coping methods, and react through appropriate activities. Therefore, personal interpretations and reactions may lead to different changes and consequences in an environment. Based on this perspective, an individual’s psychological flexibility can influence their response to a stressful event, which, in turn, can influence the dynamics of their specific relationships and overall family functioning.

In a family with IC, the conflict arising from the co-parenting process with grandparents can be a stressful event for parents. Further, parents’ psychological flexibility levels can determine how they handle such stressful events and influence the fundamental interactions in the family (e.g., co-parent dyad, parent–child dyad). For example, in the face of unsupportive IC, parents with high psychological flexibility levels can assess their own and their counterparts’ behaviors in a non-judgmental manner. Consequently, they can effectively distance themselves from negative emotions and create a conducive environment to mitigate conflict [46,48]. Conversely, parents with low psychological flexibility levels are emotionally vulnerable to conflict [41,46] and may respond in reactive and aggressive ways. For example, they may blame grandparents for undermining their parenting efforts, or they may increase their authoritarian parenting style for children to emphasize authority. This, in turn, may exacerbate unsupportive IC, family conflicts, and children’s problematic behaviors [26]. Thus, parents’ psychological flexibility can influence the underlying contexts of specific family dynamics [46]. These contrasting psychological flexibility levels are likely to buffer or exacerbate the negative influence of unsupportive IC on parenting styles and children’s SC.

### 1.4. The Current Study

**Figure 1:**
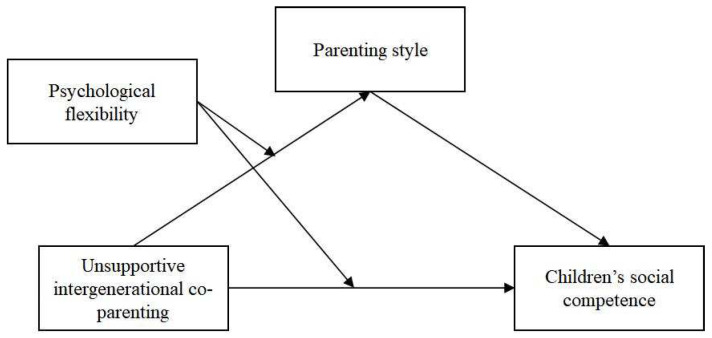
The proposed moderated mediation model.

The primary goal of this study is to provide an explanatory mechanism for the relationship between unsupportive IC and children’s SC by testing the mediating effect of parenting styles and the moderating effect of parents’ psychological flexibility (see Figure 1). Mothers and grandparents often share most of the childcare work in multigenerational Chinese families [49]. Thus, this study is interested in mother–grandparent IC. Based on the previous studies and theories, this study hypothesizes that:

(a) Unsupportive IC is negatively associated with children’s SC.
(b) Parenting style plays a mediating role in the association between unsupportive IC and children’s SC. Specifically, unsupportive IC is associated with less authoritative parenting and more authoritarian parenting, which are associated with less SC in children.
(c) Maternal psychological flexibility moderates the association between unsupportive IC and parenting style as well as the direct association between unsupportive IC and children’s SC. Therefore, the negative effects of unsupportive IC on parenting style, which decreases authoritative parenting and increases authoritarian parenting, will be significantly reduced for mothers with high psychological flexibility in contrast to mothers with low psychological flexibility. Similarly, the negative influence of unsupportive IC on children’s SC will be significantly reduced for mothers with high psychological flexibility. Because different levels of psychological flexibility may buffer or exacerbate the negative impact of unsupportive IC on parenting styles and children’s SC, we further hypothesized that mothers with high psychological flexibility may function better than others with regard to parenting styles and children’s SC when unsupportive IC is low, and mothers with low psychological flexibility may function more poorly than others with regard to parenting styles and children’s SC when unsupportive IC is high.

## 2. Materials and Methods

### 2.1. Participants

Participants were 412 children (201 boys and 211 girls) aged 3–6 from four public kindergartens in the urban area of Shanghai, China, and their mothers. Children’s mean age was 4.55 years (SD = 0.91). Of the mothers, 3.6% had a high school education or below, 21.4% attended professional or technical school, 51.7% had an undergraduate degree, and 23.3% held a graduate degree or above.

### 2.2. Procedures

The mothers were asked to complete an online survey. This survey comprised multiple questionnaires that measured self-reported IC, psychological flexibility, parenting styles, and children’s SC. The study was approved by the Research Ethics Committee at the authors’ institution. Written consent was obtained from all participating children and their mothers.

### 2.3. Measures

#### 2.3.1. Unsupportive IC

Seven items used in prior research [24] and covering a range of unsupportive IC (e.g., “My partner competes with me for the child’s attention”) were adapted for this study. A 5-point Likert scale was adopted (1 = never to 5 = always). The mean of the seven items was taken, with higher scores indicating more unsupportive IC. Cronbach’s α for these items was 0.86.

#### 2.3.2. Psychological Flexibility

We measured maternal psychological flexibility using the Acceptance and Action Questionnaire-II [50]. Mothers were requested to respond to 10 self-statements (e.g., “It’s OK if I remember something unpleasant”, “Emotions cause problems in my life”) using a 7-point Likert-type scale (1 = never true to 7 = always true). Items on psychological inflexibility were reverse-coded. The mean score of the 10 items was calculated, with higher scores indicating greater psychological flexibility. Cronbach’s α in the study was 0.80.

#### 2.3.3. Parenting Styles

Twenty-six items were adopted from the Parenting Styles and Dimensions Questionnaire [51] to measure authoritative and authoritarian parenting. Previous studies have demonstrated that this measure achieved good reliability and validity in Chinese samples [52]. The authoritative subscale contains 15 items (e.g., “Shows patience with child”), and the authoritarian subscale contains 11 items (e.g., “Scolds and criticizes to make child improve”). Mothers rated each item using a 5-point Likert-type scale (1 = never to 5 = always). The mean of the scores for each subscale’s items were computed, and higher scores indicated higher levels of authoritative and authoritarian parenting. Cronbach’s α was 0.91 and 0.89 for mothers on the authoritative and authoritarian parenting style subscales, respectively.

#### 2.3.4. Children’s SC

This scale was developed by LaFreniere and Dumas [53] to assess children’s SC. Earlier studies have demonstrated that it has good reliability and validity in China [54]. This study used the 10-item SC subscale (e.g., “Negotiates solutions to conflicts with other children”), rated on a 6-point Likert scale (1 = never to 6 = always). The mean of the items was calculated, and a higher score indicated a greater level of SC. Cronbach’s α for the SC subscale was 0.89 in this study.

### 2.4. Data Analysis

We computed the descriptive statistics and bivariate correlations of the study variables, and then we examined the mediation model and the moderated mediation model using Hayes’ PROCESS macro for SPSS [55]. The predictors were all standardized as Z scores prior to analyses. The method provided the direct, total, and indirect effects of an independent variable on a dependent variable. Based on 5000 bootstrapped samples, 95% of the deviation is used to correct the confidence interval. If the 95% confidence intervals (CI) include zero, the tested effect is not significant [56].

## 3. Results

### 3.1. Preliminary Analysis

The missing data rate on the study variables was 0–22.60%. The result of Little’s MCAR test was χ^2^ =19.35, df = 12, *p* = 0.08, indicating that data were missing completely at random. We handled the missing data using maximum likelihood estimation [57].

Descriptive statistics and bivariate correlations of the study variables are presented in Table 1. The results showed that child age was positively associated with SC, indicating that children’s SC improves with age. Child gender was not significantly associated with any variables of interest in this study. Mothers’ education was negatively related to unsupportive IC and maternal authoritarian parenting, and positively related to maternal authoritative parenting, psychological flexibility, and children’s SC. Thus, child age and mother’s education were statistically controlled in the following analyses.

Unsupportive IC was negatively correlated with children’s SC and maternal authoritative parenting, and positively correlated with maternal authoritarian parenting. Maternal authoritative parenting was positively correlated with children’s SC, while maternal authoritarian parenting was negatively correlated with children’s SC. In addition, maternal psychological flexibility was negatively correlated with unsupportive IC and maternal authoritarian parenting, and positively correlated with maternal authoritative parenting and children’s SC.

### 3.2. Testing for Mediation Effect

Model 4 in the PROCESS macro [55] was used to examine whether maternal authoritative and authoritarian parenting mediated the relation between unsupportive IC and children’s SC. As hypothesized, unsupportive IC negatively predicted maternal authoritative parenting (β = −0.33, SE = 0.04, *p* < 0.001) and positively predicted maternal authoritarian parenting (β = 0.40, SE = 0.03, *p* < 0.001). After authoritative and authoritarian parenting were entered, the negative effect of unsupportive IC on children’s SC was not statistically significant (β = 0.00, SE = 0.05, *p* = 0.99). Maternal authoritative parenting positively predicted children’s SC (β = 0.54, SE = 0.07, *p* < 0.001), and maternal authoritarian parenting negatively predicted children’s SC (β = −0.16, SE = 0.07, *p* = 0.03). The specific regression model parameters are presented in Table 2.

The total effect of unsupportive IC on children’s SC was statistically significant (β = −0.16, SE = 0.03, 95% CI = [−0.218, −0.116]). The indirect effects of unsupportive IC on children’s SC through maternal authoritative parenting (β = −0.12, SE = 0.02, 95% CI = [−0.174, −0.085]) and authoritarian parenting (β = −0.04, SE = 0.02, 95% CI = [−0.086, −0.003]) were both significant. The direct effect of unsupportive IC on children’s SC was not significant (β = 0.00, SE = 0.04, 95% CI = [−0.072, 0.724]). Therefore, mothers’ authoritative and authoritarian parenting fully mediated the relationship between unsupportive IC and children’s SC.

### 3.3. Testing for Moderated Mediation

Maternal psychological flexibility was expected to moderate the first stage of the indirect associations as well as the direct associations between unsupportive IC and children’s SC. The PROCESS macro (Model 8) was used to examine this potentially moderated mediation model. The results are shown in Table 3. Maternal psychological flexibility had significant effects on maternal authoritative (β = 0.26, SE = 0.05, t = 6.38, *p* < 0.001) and authoritarian parenting (β = −0.28, SE = 0.04, t = −6.49, *p* < 0.001). Moreover, significant interactions between unsupportive IC and psychological flexibility were found on maternal authoritative (β = 0.11, SE = 0.04, t = 3.02, *p* = 0.003) and authoritarian parenting (β = −0.19, SE = 0.04, t = −5.29, *p* < 0.001), but not on children’s SC (β = 0.04, SE = 0.04, t = 1.04, *p* = 0.30). The results of bias-corrected percentile bootstrapping further showed that the indirect effect of unsupportive IC on children’s SC through maternal authoritative parenting was moderated by maternal psychological flexibility (index of moderated mediation = 0.04, SE = 0.03, 95% CI = [0.003, 0.108]) and the indirect effect of unsupportive IC on children’s SC through maternal authoritarian parenting was moderated by maternal psychological flexibility (index of moderated mediation = 0.02, SE = 0.01, 95% CI = [0.002, 0.050]).

**Figure 2:**
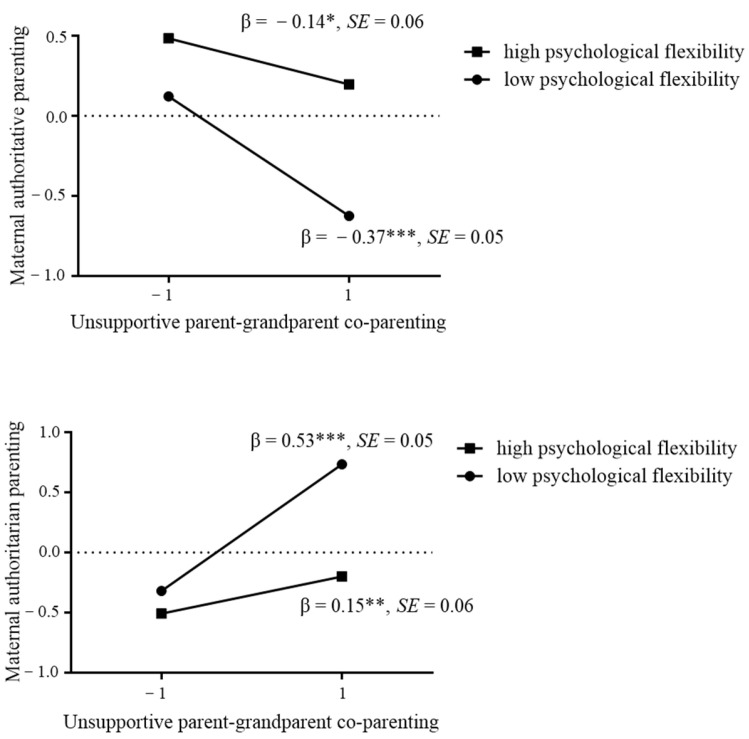
Interactions between unsupportive intergenerational co-parenting and maternal psychological flexibility. * *p* < 0.05, ** *p* < 0.01, *** *p* < 0.001.

To understand the nature of the interactions, we further conducted simple slopes for maternal authoritative and authoritarian parenting on unsupportive IC at a low value and a high value (one SD below and above the mean) of maternal psychological flexibility (Figure 2). The results indicated that unsupportive IC was significantly related to authoritative parenting for mothers with low flexibility (βsimple = −0.37, SE = 0.05, t = −6.81, *p* < 0.001) and high psychological flexibility (βsimple = −0.14, SE = 0.06, t = −2.26, *p* = 0.02), and the magnitude of the association was greater for mothers with low psychological flexibility. Additionally, unsupportive IC was significantly related to authoritarian parenting for mothers with low (β = 0.53, SE = 0.05, t = 10.36, *p* < 0.001) and high psychological flexibility (β = 0.15, SE = 0.06, t = 2.60, *p* = 0.009), and the magnitude of the association was greater for mothers with low psychological flexibility.

Furthermore, following the procedure suggested by Gaudreau et al. [58], we compared the predicted values under four conditions: high unsupportive IC—high maternal psychological flexibility (HH), high unsupportive IC—low maternal psychological flexibility (HL), low unsupportive IC—high maternal psychological flexibility (LH), and low unsupportive IC—low maternal psychological flexibility (LL). The results showed that mothers with LH scored higher on maternal authoritative parenting and lower on maternal authoritarian parenting than others, and mothers with HL scored lower on maternal authoritative parenting and higher on maternal authoritarian parenting than others. Additionally, mothers with HH and LL had no significant differences on maternal authoritative and authoritarian parenting, and mothers with LH and LL had no significant differences on maternal authoritarian parenting.

## 4. Discussion

This study employed a mediation model to examine whether unsupportive IC was indirectly associated with children’s SC through maternal parenting styles. This study also analyzed whether the first stage of the indirect association, as well as the direct association, was moderated by maternal psychological flexibility. The findings deepen the understanding of the extent to which unsupportive IC impacts children’s SC.

### 4.1. The Mediating Role of Parenting Styles

Consistent with the proposed hypotheses, unsupportive IC was negatively correlated with children’s SC. Maternal authoritative and authoritarian parenting styles played mediating roles in the relationship, extending the previous theory and empirical research [17,18]. This result also advances the understanding of family systems theory based on its application to family subsystems (e.g., coparenting and parenting subsystems) and child adjustment. While family systems theory has been verified by various studies [37,59], the current study is novel as it considers its applicability in the IC field and demonstrates a negative spillover effect on parenting styles and children’s SC. Although previous studies have ascertained IC as a critical factor that influences child outcomes [16,28], the current study presents the first empirical attempt that focuses on the negative impact of unsupportive IC on children’s SC and shows the mediating role of parenting styles.

Further, the results of the first stage of the mediating process are of note, besides the the overall mediating effect. The results revealed that unsupportive IC was negatively and positively related to maternal authoritative and authoritarian parenting, respectively. These findings support the theoretical view of the relationship between the co-parenting and parenting subsystems [7,17]. Taking care of grandchildren is a crucial form of support provided by grandparents to help reduce parenting stress [60]. However, mothers who perceive grandparents as unsupportive in an IC relationship may experience high parenting stress, which is associated less with authoritative parenting and more with authoritarian parenting [61,62]. Further, unsupportive IC can impair family functioning and lead to increased levels of conflict in intergenerational relationships [26], disrupting maternal parenting behaviors and practices [63]. In China, conflict regarding child-rearing often emerges between parents and grandparents [19,23]. However, Chinese families place significant emphasis on filial piety and require their children and grandchildren to obey, respect, and take care of their elders [64]. Thus, while parents may not directly and explicitly express their anger and hostility, a spillover effect may occur in their parenting behaviors toward their children.

### 4.2. The Moderating Role of Psychological Flexibility

This study examined the moderating role of maternal psychological flexibility on the first stage of the indirect association, as well as the direct association, between unsupportive IC and children’s SC. The results showed that maternal psychological flexibility did not moderate the relationship between unsupportive IC and children’s SC. Thus, unsupportive IC directly negatively affected children’s SC, regardless of the level of maternal psychological flexibility. The current study emphasizes that a family is a complex system with multiple functional levels that involve interactions between the co-parent dyad and parent–child dyad. Thus, maternal psychological flexibility traits cannot directly affect the relationship between the IC subsystem (e.g., unsupportive co-parenting) and children’s adjustment (e.g., SC). However, maternal psychological flexibility can affect the relationship between the IC subsystem and the parent–child subsystem (e.g., parenting styles).

The results further revealed that unsupportive IC had a significant negative association with authoritative parenting and a positive association with authoritarian parenting for mothers with low and high psychological flexibility. Further, the magnitudes of these associations were higher for mothers with low psychological flexibility. These results suggest that unsupportive IC has a stronger negative impact on the parenting behaviors of mothers with low psychological flexibility, as mothers with high psychological flexibility appear less susceptible to unsupportive IC. These findings are consistent with the VSA [45] and transactional stress models [47], which suggest that personal characteristics and vulnerabilities may buffer or exacerbate the negative effects of stressful events on individual and family functioning. Maternal psychological flexibility refers to the ability to accept negative emotional experiences while participating in value-based behaviors [41]. The results showed that maternal psychological flexibility could act as a buffer to help reduce the adverse influence of unsupportive IC on parenting styles.

The results of the bi-condition tests indicated that mothers with high psychological flexibility in low unsupportive IC relationships scored higher for authoritative parenting and lower for authoritarian parenting than mothers with other conditions. Mothers with low psychological flexibility in highly unsupportive IC relationships scored lower for authoritative parenting and higher for authoritarian parenting than mothers with other conditions. This confirms that the advantages of high psychological flexibility are present not only in situations when unsupportive IC is high, but also when unsupportive IC is low. Prior studies have also indicated that mothers with higher psychological flexibility exhibit higher parenting-specific psychological flexibility [44]. Further, higher parenting-specific psychological flexibility has been positively related to the greater use of positive parenting strategies and behaviors [42].

### 4.3. Limitations and Future Directions

This study reveals how unsupportive IC is related to children’s SC. However, there are several limitations that should be addressed in future research. First, this study was correlational, precluding the examination of causality or directionality. Although the ecological model of co-parenting [17] suggests that co-parenting may affect parenting styles and children’s SC, parenting styles may also be affected by children’s adjustment [5] and co-parenting between primary caregivers [65]. Thus, a longitudinal design is required to delineate the direction of the relationships between IC, parenting styles, and children’s SC.

Second, while self-reports of unsupportive IC, parenting styles, and psychological flexibility can provide relevant information, future studies should collect multiple-source data to control for social desirability. In the co-parenting context, collecting reports from both sides of the co-parenting dyad would be pertinent for reducing reporting bias. Similarly, children’s SC was assessed by mothers’ self-reporting, which may also be affected by social desirability. In future studies, it would be helpful to collect evaluations from both parents or from others who have close contact with the children. An evaluation made by external observers, such as the teachers at the kindergartens attended by children, could provide less-biased data.

Third, the family power structure determines who is more entitled to make decisions, allocate resources, and manage conflicts in the family system [66], which may potentially affect family functioning in the context of child-rearing practices [67]. The spillover effect of mothers’ perceived unsupportive IC on parenting styles could be more pronounced in grandparent-dominated households than in mother-dominated households. Thus, future studies should consider the moderating role of the family power structure in three-generation families.

Fourth, only mothers were included in this study. Fathers’ perceived co-parenting with grandparents may also serve as a key component of the family system in three-generational families and be related to child development [68]. Chinese fathers and mothers play different roles in children’s socialization and interact with children in different ways [69]. Mothers serve mainly as source of emotional support, maternal warmth, and affection, whereas fathers are considered to be the authority figure in the family to help children learn societal values and develop appropriate behaviors. Previous studies have found that maternal warmth significantly predicted children’s emotional adjustment, while paternal warmth significantly predicted their social adjustment [70]. It is possible that fathers’ perceived unsupportive IC is more likely to spill over to the socialization of children through their parenting styles, and the moderating role of paternal psychological flexibility may be more obvious. Thus, the results of the present study may not be generalizable to fathers. It is also important to investigate the influence of fathers’ perceived unsupportive IC on children’s SC and its mechanisms in co-parenting families.

Finally, the generalizability of this study should be taken with caution, as it is uncertain whether these findings, based on an urban Chinese sample, can be generalized to rural areas or other societies. Globally, it has become increasingly common for grandparents to be involved in child-rearing. However, grandparents’ motivations and ways of caring for children, as well as parental attitudes and responses to IC conflicts, may vary across cultures and societies [23]. Further clarity is also required in terms of the generalizability of this study’s findings to related outcomes (e.g., theory of mind, executive function) and more specific dimensions of parenting (e.g., psychological control). Future research should be conducted across diverse societies and regions, and should consider incorporating more child development outcomes and parent-specific parenting behaviors.

### 4.4. Practical Implications

Despite the above limitations, this study presents several practical implications. First, an unsupportive IC relationship is a risk factor for children’s SC. Thus, parents and educators should pay careful attention to the social adjustment of children from IC families. Moreover, unsupportive IC relationships can set a negative precedent for young children’s social interactions. Thus, a harmonious and supportive family atmosphere should be promoted to mitigate or prevent social maladjustment (e.g., low SC) in preschool children.

Second, this study demonstrates the mediating role of parenting styles in the relationship between unsupportive IC and children’s SC. This finding may benefit practitioners in understanding and determining the pathways that link unsupportive IC to young children’s SC. Further, it may be necessary to improve children’s SC through mothers who perceive highly unsupportive IC by reducing maternal authoritarian parenting and increasing maternal authoritative parenting.

Third, this study reveals that maternal psychological flexibility plays a moderating role in the indirect relationship between unsupportive IC and children’s SC. This finding suggests that mothers with low psychological flexibility should be prioritized in interventions because of their high and low levels of authoritarian and authoritative parenting, respectively. Mothers with low psychological flexibility may benefit from preventive interventions based on acceptance and commitment therapy [71] in order to maintain an enhanced and non-judgmental awareness of their experiences and mitigate the negative influences from external circumstances or events to act harmoniously with their parenting values [44].

## 5. Conclusions

This study explored the mediating role of parenting styles and the moderating role of maternal psychological flexibility in the relationship between unsupportive IC and children’s SC. It found that parenting styles could serve as a potential mechanism to link unsupportive IC to SC. Unsupportive IC was negatively associated with children’s SC through decreased authoritative parenting and increased authoritarian parenting. Moreover, the first stage of the mediation mechanism was moderated by maternal psychological flexibility. The negative influence of unsupportive IC on maternal parenting styles was greater for mothers with lower psychological flexibility.

## Figures and Tables

**Table 1:** Descriptive statistics and correlations among observed variables.

	M	SD	1	2	3	4	5	6	7	8
1 Child age	4.55	0.91	1							
2 Child gender	0.51	0.50	−0.00	1						
3 Mother education	2.95	0.77	−0.10 *	−0.09	1					
4 Unsupportive IC	1.86	0.67	0.00	0.00	−0.12 **	1				
5 Maternal authoritative parenting	4.21	0.56	0.02	0.00	0.20 ***	−0.42 ***	1			
6 Maternal authoritarian parenting	1.79	0.54	0.04	0.04	−0.16 **	0.51 ***	−0.53 ***	1		
7 Maternal psychological flexibility	5.40	0.90	0.04	−0.04	0.18 ***	−0.39 ***	0.45 ***	−0.48 ***	1	
8 Children’s SC	4.12	0.72	0.11 *	0.05	0.09	−0.24 ***	0.49 ***	−0.34 ***	0.34 ***	1

Note. Child gender: 0 = boys, 1 = girls. IC = intergenerational co-parenting, SC = social competence. * *p* < 0.05, ** *p* < 0.01, *** *p* < 0.001.

**Table 2:** The mediating role of maternal authoritative and authoritarian parenting.

	Maternal Authoritative Parenting			Maternal Authoritarian Parenting			Children’s SC		
	β	SE	t	β	SE	t	β	SE	t
Child age	0.04	0.04	0.83	0.03	0.04	0.76	0.07	0.03	2.39 *
Mother education	0.16	0.04	3.51 ***	−0.10	0.04	−2.33 *	−0.00	0.03	−0.04
Unsupportive IC	−0.40	0.04	−8.98 ***	0.50	0.04	11.64 ***	0.00	0.04	0.01
Maternal authoritative parenting							0.30	0.04	8.07 ***
Maternal authoritarian parenting							−0.09	0.04	−2.22 *
*R* ^2^	0.20			0.27			0.25		
*F*	34.07 ***			50.21 ***			27.75 ***		

Note. IC = intergenerational co-parenting, SC = social competence. * *p* < 0.05, *** *p* < 0.001.

**Table 3:** The mediated moderating effect of unsupportive intergenerational co-parenting and social competence.

	Maternal Authoritative Parenting			Maternal Authoritarian Parenting			Children’s SC		
	β	SE	t	β	SE	t	β	SE	t
Child age	0.03	0.04	0.77	0.03	0.04	0.73	0.10	0.04	2.32 *
Mother education	0.11	0.04	2.62 **	−0.05	0.04	−1.33 *	−0.01	0.04	−0.23
Unsupportive IC	−0.26	0.05	−5.65 ***	0.34	0.04	8.01 ***	0.02	0.05	0.40
Psychological flexibility	0.30	0.05	6.38 ***	−0.28	0.04	−6.49 ***	0.12	0.05	2.30 *
Interaction	0.11	0.04	3.02 **	−0.19	0.04	−5.29 ***	0.04	0.04	1.04
Maternal authoritative parenting							0.39	0.05	7.28 ***
Maternal authoritarian parenting							−0.07	0.06	−1.31
*R* ^2^	0.30			0.40			0.27		
*F*	34.99 ***			53.34 ***			21.02 ***		

Note. IC = intergenerational co-parenting, SC = social competence. * *p* < 0.05, ** *p* < 0.01, *** *p* < 0.001.

## Data Availability

The data presented in this study are available on request from the corresponding author. The data are not publicly available due to the fact that personal information was gathered.
